# Mapping of meiotic recombination in human preimplantation blastocysts

**DOI:** 10.1093/g3journal/jkad031

**Published:** 2023-02-03

**Authors:** Yuanlin Ma, Jing Wang, Rong Li, Chenhui Ding, Yan Xu, Canquan Zhou, Yanwen Xu

**Affiliations:** Reproductive Medicine Center, The First Affiliated Hospital of Sun Yat-sen University, Guangzhou, Guangdong 510080, China; The Key Laboratory for Reproductive Medicine of Guangdong Province, Guangzhou 510080, China; Reproductive Medicine Center, The First Affiliated Hospital of Sun Yat-sen University, Guangzhou, Guangdong 510080, China; The Key Laboratory for Reproductive Medicine of Guangdong Province, Guangzhou 510080, China; Reproductive Medicine Center, The First Affiliated Hospital of Sun Yat-sen University, Guangzhou, Guangdong 510080, China; The Key Laboratory for Reproductive Medicine of Guangdong Province, Guangzhou 510080, China; Reproductive Medicine Center, The First Affiliated Hospital of Sun Yat-sen University, Guangzhou, Guangdong 510080, China; The Key Laboratory for Reproductive Medicine of Guangdong Province, Guangzhou 510080, China; Reproductive Medicine Center, The First Affiliated Hospital of Sun Yat-sen University, Guangzhou, Guangdong 510080, China; The Key Laboratory for Reproductive Medicine of Guangdong Province, Guangzhou 510080, China; Reproductive Medicine Center, The First Affiliated Hospital of Sun Yat-sen University, Guangzhou, Guangdong 510080, China; The Key Laboratory for Reproductive Medicine of Guangdong Province, Guangzhou 510080, China; Reproductive Medicine Center, The First Affiliated Hospital of Sun Yat-sen University, Guangzhou, Guangdong 510080, China; The Key Laboratory for Reproductive Medicine of Guangdong Province, Guangzhou 510080, China

**Keywords:** Han Chinese, human, blastocyst, homologous recombination, meiosis, reciprocal translocation

## Abstract

Recombination is essential for physical attachments and genetic diversity. The Han Chinese population is the largest ethnic group worldwide, therefore, the construction of a genetic map regarding recombination for the population is essential. In this study, 164 and 240 couples who underwent preimplantation genetic testing for monogenic diseases or segmental rearrangement were included in the analysis. Blastocysts and probands from couples who underwent preimplantation genetic testing for monogenic diseases by single nucleotide polymorphism array were included for recombination analysis. The location of recombination was determined from haplotype phase transitions in parent-offspring pairs at loci where the parents were heterozygous. The genetic map for Chinese *in vitro* fertilization embryos was constructed by the expectation–maximization algorithm with chip-level data. Our results confirmed that homologous recombination occurred more often in maternal chromosomes, and the age effect was more significant in maternal homologous recombination. A total of 6,494 homologous recombination hotspots (32.3%) were identified in genes of Online Mendelian Inheritance in Man. A uniform association between homologous recombination and aneuploidy was not established. In addition, carriers with identified breakpoints of reciprocal translocations were analyzed, and locations of breakpoints were found partly overlapped with homologous recombination hotspots, implying a possible similar mechanism behind both events. This study highlights the significance of constructing a recombination map, which may improve the accuracy of haplotype analysis for preimplantation genetic testing for monogenic diseases. Overlapping locations of translocation and recombination are worthy of further investigation.

## Introduction

During meiosis, recombination between paired homologous chromosomes contributes to genetic diversity by introducing new allele combinations (Chen *et al.* 2020). Recombination is essential for forming physical attachments between homologous chromosomes, which is beneficial for accurate chromosome segregation during meiosis. Meiotic recombination is initiated by the form of double-strand breaks (DSBs) catalyzed by SPO11 (Keeney *et al.* 1997). DSB formation is nonrandom, with certain regions more liable to form DSBs (Halldorsson *et al.* 2019). After a large amount of DSBs are formed, proteins essential for repairing DSBs will be activated, such as MRE11, RAD50, NBS1, EXO1, DNA2 (Garcia *et al.* 2011), RNA polymerase III (Liu *et al.* 2021), RAP, and RAD5 (Gray *et al.* 2018; Gray and Cohen 2016). Most DSBs can be repaired by homologous recombination (HR), resulting in a crossover or noncrossover (Halldorsson *et al.* 2019). Other pathways, such as nonhomologous end-joining (NHEJ), microhomology-mediated end-joining, and single-strand annealing, also participate in DNA repairment, leading to the possibility of chromosomal abnormalities, such as reciprocal translocation and aneuploidy. Once synaptonemal complex assembly, the structural basis for HR, cannot be completed, nonobstructive azoospermia and premature ovarian failure may happen (Sánchez-Sáez *et al.* 2020).

Given its critical role in biological and evolutionary processes, the occurrence of recombination might be expected to be conservative across different populations. Yet, tremendous heterogeneity in the rates and patterns of recombination persists among individuals, sexes, and populations (Stapley *et al.* 2017). Nowadays, most genetic maps have been constructed using almost individuals of European or African ancestry (Weber 2002). To the best of our knowledge, only one study focused on Asians (Korean and Mongolian pedigrees) was published (Bleazard *et al.* 2013). The Han Chinese population is the largest ethnic group worldwide, therefore, the construction of a genetic map is essential and valuable.

One way to learn human meiotic recombination is to estimate the locations of crossovers from genomic segments shared among relatives (Kong *et al.* 2010; Halldorsson *et al.* 2019) or linkage-disequilibrium patterns in populations (Vergara-Lope *et al.* 2019). However, only a few gametes per individual generate offspring. This “missing data” problem is significant in understanding what decided the embryos to be sifted out. Theoretically, the most powerful approach to studying meiosis is observing meiotic processes directly in gametes (Lynn *et al.* 2002; Ioannou *et al.* 2019). Genotyping or sequencing of gametes would provide detailed information about recombination (Ottolini *et al.* 2015; Bell *et al.* 2020). However, the scarcity of human oocytes limits the observation of gametes.

In preimplantation genetic testing for monogenic diseases (PGT-M), DNA from biopsied cells of preimplantation embryos is available, which may provide valuable genetic information for meiotic recombination (Konstantinidis *et al.* 2020). The haplotyping method for PGT-M provides novel insights into the genome dynamics of preimplantation development (Tšuiko *et al.* 2021). Using siCHILD/haplarithmisis (Tšuiko *et al.* 2021) or Karyomapping (Konstantinidis *et al.* 2020), copy number variance (CNV) origin and happening time could be inferred to understand the mechanisms underlying aneuploidy formation. It remains unknown whether the recombination pattern is related to the occurrence of aneuploidies and whether the specific aneuploid chromosome introduces changes in the recombination pattern of others.

The molecular mechanism underlying the formation of chromosomal translocation also remains elusive. However, lots of studies focused on oncogenic translocation, with the discovery that DNA topoisomerase II-associated translocations are the main driver of some common hematological and solid tumors (Gómez-Herreros 2019). It was supposed that DSB formation was also essential for reciprocal translocation (Gómez-Herreros 2019; Chen *et al.* 2020). It is interesting to investigate the relationship between recombination and reciprocal translocation.

This study described a genetic map from preimplantation embryos by trio-based methods using samples from biopsied embryos and related individuals. The possible relevant factors of HR and hotspots in human blastocysts were studied, and hotspots were identified in genes of Online Mendelian Inheritance in Man (OMIM). In addition, locations of breakpoints of reciprocal translocation were determined, and overlapping locations with those of HR hotspots were analyzed. Our study may provide further evidence to support the hypothesis that reciprocal translocations occur more often in HR hotspots.

## Materials and methods

### Ethics statement

Institutional review board approval was obtained for this study by the institutional review board of the First Affiliated Hospital of Sun Yet-Sen University. All studies were conducted in accordance with the Helsinki Declaration. Informed consent was waived due to the retrospective analysis of anonymized data. All primary data were encoded such that informative single nucleotide polymorphisms (SNPs) were represented as A and B. Only secondary data with informative SNPs coded as A and B were used for data analysis.

### Study population

This retrospective study included couples for PGT-M by Karyomapping at the Reproductive Medicine Center of the First Affiliated Hospital of Sun Yet-Sen University from 2016 November 1 to 2019 December 31 to construct the genetic map. Patients with reciprocal translocation were also included for PGT for segmental rearrangement (PGT-SR) from 2017 August 1 to 2019 December 31. All karyotypes of couples and probands in PGT were normal, except for reciprocal translocation carriers. Only the Han Chinese population was included. Exclusion criteria were as follows: (1) no embryo available for biopsy, (2) no euploid embryo after PGT-M, (3) breakpoint of reciprocal translocation could not be identified, and (4) embryos with a call rate of <0.9 or allele drop out of >0.05, indicating poor whole-genome amplification (WGA).

### Controlled ovarian stimulation

Controlled ovarian stimulation was performed according to routine protocols (Lu *et al.* 2020). Intracytoplasmic sperm injection (ICSI) was used in all cases for PGT, aiming to eliminate the contamination risk from extraneous sperm DNA. Oocytes displaying 2 pronuclei (2PN) were cultured in separate microdrops up to the blastocyst stage according to the protocol of sequential culture in a humidified atmosphere (5% O_2_ and 6% CO_2_). A trophectoderm biopsy was performed on expanded blastocysts on postretrieval day 5 or 6, depending on the developmental stage of individual embryos. All embryos underwent a single-blastocyst biopsy and were vitrified separately after the biopsy. As described previously, the cryotop method for vitrification was used, and warmed cycles were performed according to the routine protocol (Lu *et al.* 2020).

### WGA and PGT

Biopsied samples were subjected to WGA using an REPLI-g Single-Cell WGA kit (Qiagen, Germany) according to the manufacturer's instructions. Embryonic WGA and parental DNA samples were used for SNP genotyping to detect chromosome status and monogenic disease (Wang *et al.* 2019; Li *et al.* 2021). Briefly, individual DNA samples for PGT-M were hybridized with HumanKaryomap-12 v2.1 BeadChip (Illumina, USA), which contains almost 300,000 SNPs with an average distance of 9.7 kb (Wang *et al.* 2019). BlueFuse (Multiv 4.5; Illumina) was used to process data files. Haplotypes were generated by reformatting and importing genotype data into an in-house transcript. Simultaneously, aneuploidy was detected visually based on the B-allele frequency of SNPs and the logR ratio defined by Illumina, with normal DNA as a reference (Wang *et al.* 2019). The detailed steps of haplotype phasing were described previously (Wang *et al.* 2019). For PGT-SR, individual DNA samples were hybridized with HumanCytoSNP-12 v2.1 BeadChip (Illumina; Li *et al.* 2021). The breakpoints of reciprocal translocation were identified using unbalanced embryos, as we described previously (Li *et al.* 2021). Based on the deletion position of translocated chromosomes in unbalanced embryos generated by the adjacent mode, the specific location of the breakpoint could be determined (Li *et al.* 2021).

### Genetic map

Locations of crossovers were determined from haplotype phase transitions in parent–proband pairs based on informative markers where the parents were heterozygous. Initially, the location was determined by the 2 closest heterozygous markers to the crossover, providing upper and lower bounds for the location. At least 2 informative SNPs were required to determine the true recombination event. Then, a genetic map was computed using an expectation–maximization (E–M) algorithm (Halldorsson *et al.* 2019). To analyze the association between parental age and HR, only families with at least 3 offspring were used. An association study was performed by generalized estimating equations (Liang and Zeger 1986) using the “geeglm” function in the R Package geepack (Højsgaard *et al.* 2006). The standardized recombination rate (SRR) was defined as the regional recombination rate divided by the genomic average for each sex separately. HR hotspots were defined as regions with SRR not less than 10.

### Parent-of-origin and mechanistic origin of aneuploidy

The parent-of-origin and mechanistic origin of aneuploidy were assessed according to haplotype and this study's algorithm. Theoretically, it was considered an error in meiotic prophase I with the heterogeneity in centromere and an error in mitosis with homozygosity in the whole chromosome. The rest with homozygosity in the centromere but not the whole chromosome would be considered an error in meiotic prophase II (Ariad *et al.* 2021). The parental origin of CNV could be distinguished by B-allele frequencies according to the type of chromosome loss or gain. However, this study failed to distinguish the mechanistic origin of monosomy and segmental chromosome error. Aneuploid embryos were further analyzed for their relationship with HR. All embryos of the patients with aneuploidy and euploidy were included in the analysis. This study only analyzed the recombination rate of euploid chromosomes from aneuploidies and euploidies.

## Results

### Data collection

**Fig. 1.**
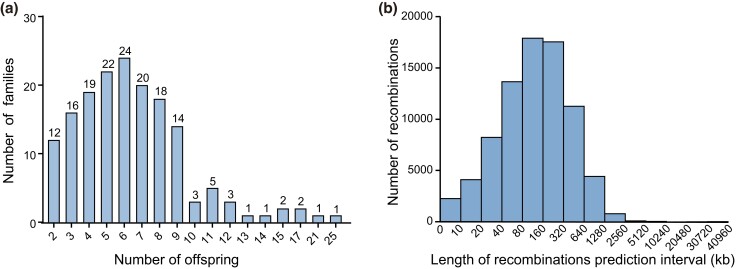
Distribution of pedigree and recombination prediction interval length. a) Pedigree distribution. Distribution of the number of families with a different sample size of offspring. All families had at least 2 offspring samples, including samples from a living offspring, terminated pregnant tissues, and/or euploid embryos generated from PGT-M cycles. b) Distribution of crossover prediction interval length. Histogram of crossover prediction intervals in all meiosis. The *x*-axis was scaled logarithmically.

A total of 194 cycles from 164 families for PGT-M by Karyomapping were included. The average maternal and paternal ages were 31.45 ± 3.95 and 33.85 ± 4.63 years per cycle, respectively. There were 142 living offspring, 10 terminated pregnant tissues with normal karyotypes, and 918 euploid embryos generated from PGT-M cycles analyzed for genetic maps (Fig. 1a). To investigate the relationship between aneuploid chromosomes and HR, 353 aneuploidy embryos were analyzed. All terminated pregnant tissues and embryos were also presented as offspring unless otherwise noted. The indications for PGT-M were β-thalassemia (*n* = 97), Duchenne muscular dystrophy (*n* = 11), hemophilia (*n* = 8), and other monogenic diseases. To investigate the relationship between translocation breakpoints and locations of HR, 240 reciprocal translocation carriers who performed PGT-SR were included in the analysis.

### Characteristics of crossover

Microarray genotype data from 293,109 SNPs allowed identifying 31,005 crossovers in 1,070 paternal meiosis and 49,466 crossovers in 1,070 maternal meiosis (Supplementary Table 1). Each offspring had an average of 75.21 crossovers. Data were quite comprehensive, including paternal crossovers between chromosomes X and Y, which were previously neglected (Coop *et al.* 2008; Hussin *et al.* 2011; Halldorsson *et al.* 2019). The median length of the recombination interval was 127,648 bp (Fig. 1b).

Supplementary Table 1 shows the amount of microarray markers used in this study and the amount of meiosis considered for computing the recombination rate and genetic maps. The crossovers found in the data from microarray-typed trios were used for computing recombination rates and genetic maps.

### Effects of parental age on the amount of recombination

**Fig. 2.**
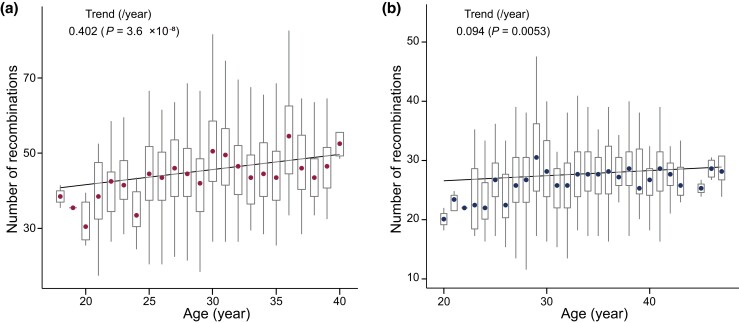
Association between parental age and amount of recombination. a) Amount of recombination *vs* the mother's age at birth. b) Amount of recombination *vs* the father's age at birth.

For maternal chromosomes, there was an age effect on the amount of recombination (Fig. 2a) that corresponded to an increase of 0.402/year (*P* = 3.6 × 10^−8^). An age-related increase in the amount of recombination (0.094/year; *P* = 0.0053) was observed in paternal chromosomes (Fig. 2b). However, the *P*-value did not indicate a very strong significance.

### Genetic map

**Fig. 3.**
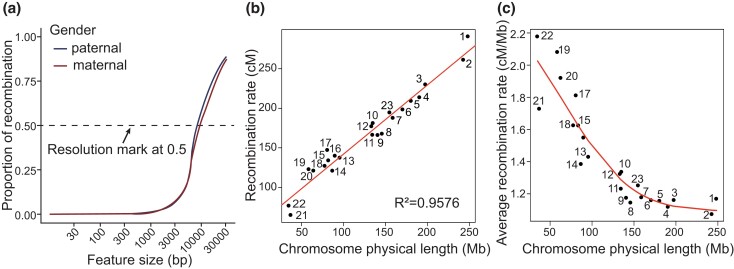
Features of recombination. a) Proportions of recombination that fell within a given feature size. b) Plot of recombination rate against physical length for 23 chromosomes. c) Plot of average recombination rate against physical length for 23 chromosomes. The number next to the point represents the chromosome number. The label on the *x*-axis represents the chromosome's physical length. The label on the *y*-axis in (b) represents the recombination rate. The label on the *y*-axis in (c) represents the average recombination rate, defined as the recombination rate divided by the chromosome's physical length.

The genetic length was 2,839.91 and 4,428.69 cM for paternal and maternal autosomal origins, respectively, corresponding to a sex-averaged length of 3,634.30 cM, as shown in Table 1. Generally, the recombination rates of maternal chromosomes were higher. The ratio of the recombination rates between maternal and paternal origins varied from 1.09 in chromosome 19 to 1.93 in chromosome 4, with an average of 1.56 in the autosomal genome. The average resolution of the genetic map was 8.622 kb, with 8.113 and 9.131 kb for paternal and maternal maps, respectively (Fig. 3a).

**Table 1.** Genetic map lengths for Han Chinese offspring.

Chr	Physical length			Genetic length (cM)		
	Begin (bp)	End (bp)	Length (Mb)	P	M	Average
1	752,566	249,195,930	248.44	213.74	367.29	290.52
2	72,184	243,020,723	242.95	194.49	327.38	260.94
3	66,894	197,839,964	197.77	168.79	290.65	229.72
4	48,283	190,777,761	190.73	145.51	281.40	213.46
5	38,139	180,704,486	180.67	151.50	266.36	208.93
6	204,909	170,885,792	170.68	147.85	248.32	198.09
7	46,239	159,107,490	159.06	148.97	225.89	187.43
8	176,818	146,288,066	146.11	119.53	215.23	167.38
9	46,587	141,027,939	140.98	131.40	200.00	165.70
10	135,708	135,429,295	135.29	137.10	224.58	180.84
11	203,788	134,937,726	134.73	136.54	195.42	165.98
12	191,619	133,770,456	133.58	145.23	208.41	176.82
13	19,263,735	115,100,353	95.84	113.74	160.37	137.06
14	20,213,937	107,274,052	87.06	97.94	143.27	120.61
15	20,161,372	102,397,317	82.24	109.91	157.38	133.65
16	105,320	90,141,477	90.04	108.32	170.75	139.54
17	18,901	81,041,338	81.02	125.14	168.50	146.82
18	12,842	77,957,325	77.94	100.09	153.36	126.73
19	267,039	59,093,464	58.83	117.20	127.76	122.48
20	63,244	62,903,830	62.84	106.64	134.77	120.71
21	10,734,842	48,084,628	37.35	55.98	73.18	64.58
22	16,114,244	51,162,059	35.05	64.30	88.41	76.36
X	93,118	155,235,833	155.14	—	194.30	—
Totals			2,944.34	2,839.91	4,622.99	3,634.30

The genetic length was calculated by dividing the total number of crossovers by total meiosis. It was assumed that the density of markers had enough power to neglect the possibility of undetected double crossovers.

Chr, chromosome; P, paternal chromosome; M, maternal chromosome.

The average recombination rate varied across individual chromosomes. Results confirmed that chromosome length was strongly correlated with recombination rate (*R*^2^ = 0.96; Fig. 3b). Although the longer chromosomes had a higher total number of recombination, the shorter ones had a higher average recombination rate. The relationship between the average recombination rate and the physical length of a chromosome could be fitted well by a smooth curve (Fig. 3c). The adjacent recombination event space was also an important feature. The average interval was smaller in maternal chromosomes than in paternal ones, except for chromosome 21 (Supplementary Table 2).

### HR hotspots

**Fig. 4.**
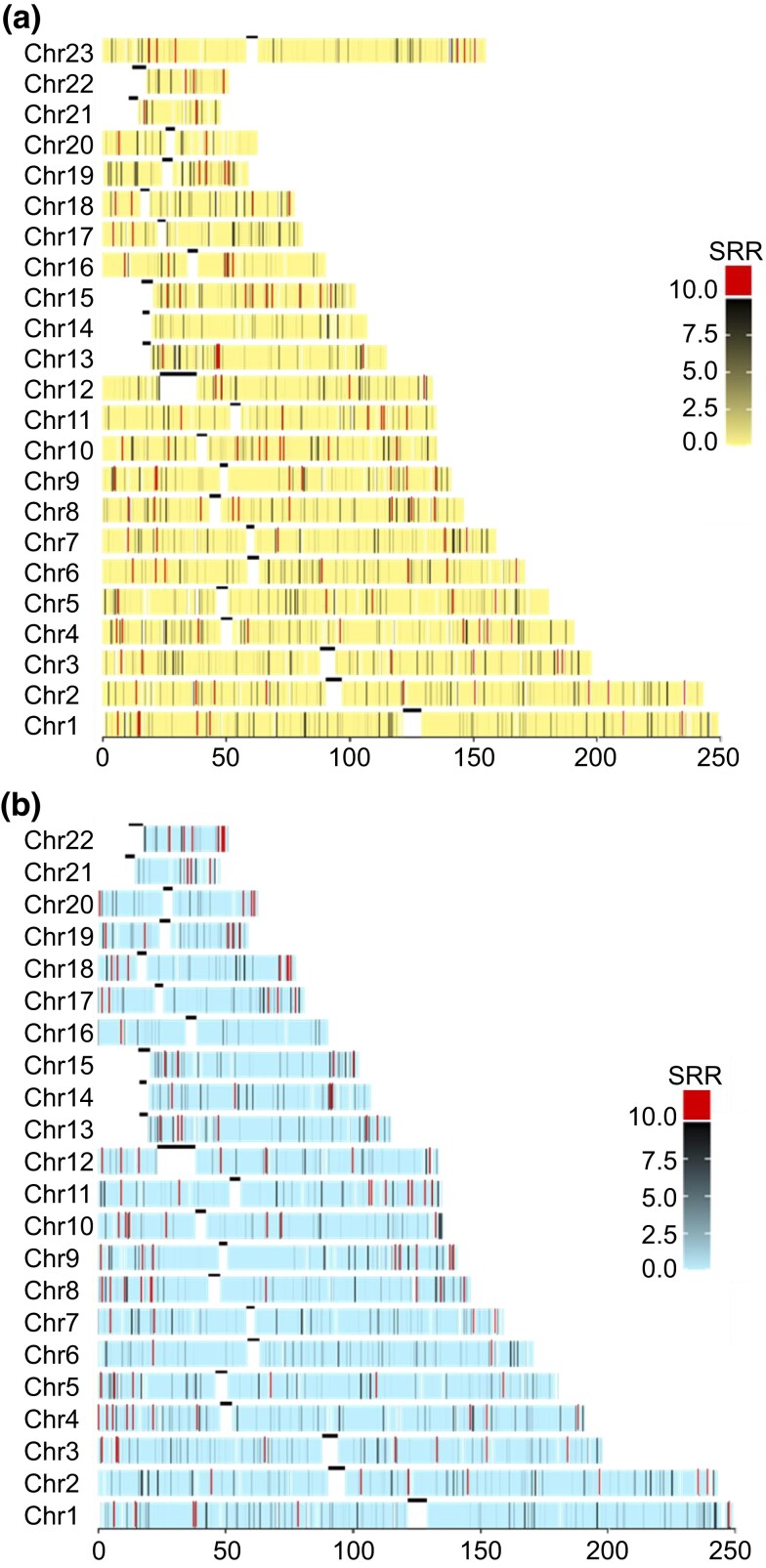
Genetic map and crossover hotspots. a) Maternal genetic map. b) Paternal genetic map. SRR is the region recombination rate divided by the genomic average. The little black line represented the centromere. There was no SNP in the short arm of the acrocentric chromosome, so no SRR was presented.

The map revealed that the number of HR hotspots (regions where the recombination rate was ≥10 times the genomic average) was different between sex-specific maps. There were 9,709 hotspots for paternal autosomes and 10,418 hotspots for maternal chromosomes (9,953 for autosomes and 465 for chromosome X; Fig. 4). Also, 4,779 hotspots overlapped within 2 sex-specific genetic maps. HR hotspots were biased toward telomere in males, whereas they were more uniformly distributed in females. There were 6,494 (32.3%) hotspots (3,152 and 3,342 from maternal and paternal hotspots, respectively) in genes of OMIM (gained on 2022 December 13), which would pose a great challenge for PGT-M (Supplementary Table 3).

### Aneuploidy and HR

Among 1,519 embryos for PGT-M, 353 were autosomal aneuploidies with 442 autosomal CNVs. More than 70% of CNVs were involved with whole chromosomes of predominantly maternal origin. Almost one-half of maternal whole chromosome gains happened during the first meiotic division, whereas most paternal ones happened during mitosis. Segmental imbalances predominantly affected paternal chromosomes, especially segmental chromosome losses (Supplementary Fig. 1). Only patients with both aneuploidies and euploidies were included for further analysis. This study failed to find a uniform association between HR and aneuploidy (Supplementary Tables 4 and 5). In aneuploidies, higher amount of recombination was found in the nonaneuploidy involved maternal chromosomes 7, 8, 12, 15, 17, 18, 19, and 20, and in the paternal chromosomes 1, 2, 4, 7, 8, 9, 14, 16, 20, 21, X and Y respectively. A more targeted comparison of genome-wide recombination events was carried out between euploid embryos and embryos determined to carry trisomy 16 alone, trisomy 21 alone, and trisomy 22 alone. We found embryos affected with trisomy 22 only (*n* = 8, *P* = 0.036) had a higher incidence of recombination compared with euploid embryos but not in trisomy 16 only (*n* = 19, *P* = 0.659) or trisomy 21 only (*n* = 3, *P* = 0.059; Supplementary Table 6).

### Breakpoints of reciprocal translocations

**Fig. 5.**
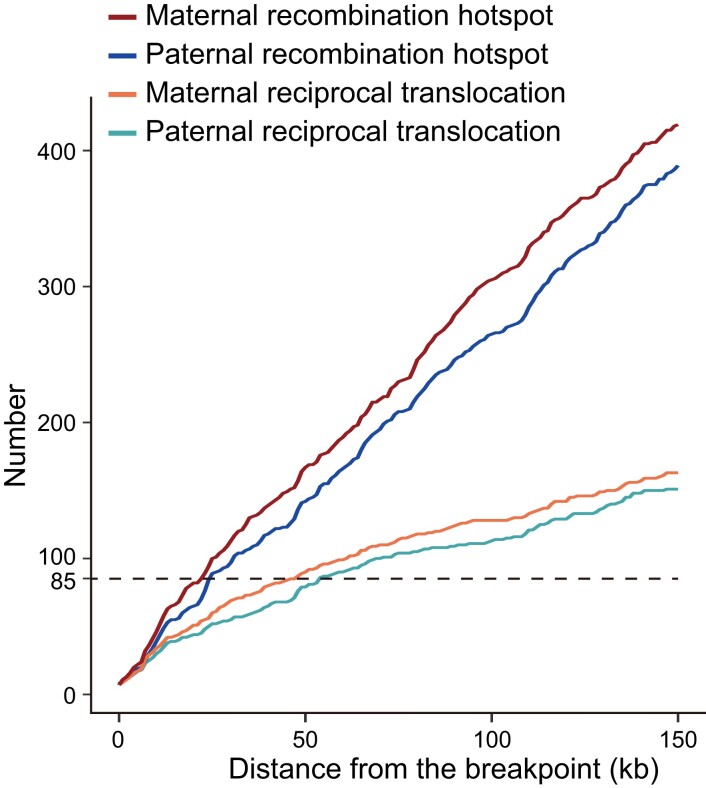
Relationship between breakpoints of reciprocal translocations and HR hotspots. Hotspots were found from the genetic maps, and breakpoints were found without distinction between paternal and maternal chromosomes.

A total of 240 reciprocal translocation carriers who performed PGT-SR were included in the analysis. The basal characteristic data, including the gender of translocation carrier, maternal age, paternal age, and the number of blastocysts biopsied, were supplemented in Table 2. Two translocation breakpoints were identified in 124 carriers, and one translocation breakpoint was identified in 92 carriers, whereas no translocation breakpoint was identified in the remaining 24 carriers. The distances between translocation breakpoints and locations of HR were calculated. It showed that 25% of translocation breakpoints (*n* = 85) overlapped with HR hotspots when the distance of the overlap was defined within 55 and 44 kb from translocation breakpoints in paternal and maternal genetic maps, respectively (Fig. 5).

**Table 2.** Basal characteristics of couples with reciprocal translocation.

Character	Translocation
Total number of couples	240
Translocation carrier (male/female)	118/122
Maternal age (years)*^a^*	30 (22–46)
Paternal age (years)*^a^*	33 (22–56)
No. of blastocysts biopsied*^a^*	8 (1–27)

Data were described as median (range).

## Discussion

This study constructed the first comprehensive genetic map of Han Chinese blastocysts and confirmed that recombination rates were affected by parental origin, parental ages, and chromosome length. There were 6,494 (32.3%) hotspots found in genes of OMIM. Interestingly, part of the breakpoints of reciprocal translocations overlapped with HR hotspots.

Crossover distribution and crossover interference were the important features of crossover. In our study, paternal and maternal chromosomes had 28.98 and 46.23 crossovers per meiosis, respectively, in accordance with the results from human gametes [26.71 and 41.6 crossovers per meiosis for sperms (Bell *et al.* 2020) and oocytes (Ottolini *et al.* 2015)], population studies from Icelanders (26.21 and 43.6 crossovers per meiosis; Halldorsson *et al.* 2019), Mongolian (27.43 and 41.96 crossovers per meiosis), and Korean (25.84 and 40.75 crossovers per meiosis; Bleazard *et al.* 2013). Recently, Konstantinidis *et al.* analyzed 389 preimplantation blastocysts from 98 couples in the United States (Konstantinidis *et al.* 2020). Karyomapping analysis through Bluefuse software was used to discover the recombination, the same methodology as our study. The number of crossovers per meiosis for paternal and maternal chromosomes was 24.0 and 41.2, respectively, similar as our study. In addition, more than 93% of recombination events in autosomal chromosomes in the study of Konstantinidis *et al.* could be found in this study.

Many factors may affect the recombination pattern, such as parental origin, parental ages, chromosome length, and so on. This study confirmed that the HR rate was higher in maternal chromosomes than in paternal ones, with a ratio of HR rates between maternal and paternal origins of >1.6 for autosomes. It could be contributed by one extra chromosome X in maternal chromosomes. A sex-specific distinction in the chromosome structure (Wang *et al.* 2021) and methylation time in germline development (Smallwood and Kelsey 2012) might also affect the amount of crossovers (Lynn *et al.* 2002; Wang *et al.* 2017). From the physiology point of view, more crossovers in maternal chromosomes may compensate for the attenuated chromosome synapsis caused by long-time exposure at the diplotene stage (Nagaoka *et al.* 2012; Cimadomo *et al.* 2018).

Age effect might affect the HR rate, especially for maternal age, consistent with previous estimates (Kong *et al.* 2004; Halldorsson *et al.* 2019). The decreased degree of chromosome agglutination (Lynn *et al.* 2002) may be due to age-related epigenetic changes (Cheung *et al.* 2018). A higher number of crossovers may protect oocytes from meiotic error (i.e. age-related maternal chromosome nondisjunction; Kong *et al.* 2004). There is also an age effect on the paternal recombination rate; however, it was weaker than the effect on the maternal recombination rate.

Chromosome length is a vital factor affecting the recombination rate, especially in maternal chromosomes. The ratio of recombination rates between maternal and paternal origins varied from 1.43 to 1.93 for chromosomes 1–12 but with lower average recombination rates, in accordance with previous study (Kong *et al.* 2002). Short chromosomes had a relatively longer axis and smaller loops (Zhang *et al.* 2014), resulting in more loops and higher crossover density (Lynn *et al.* 2002), attributed to multiple effects, such as early homolog pairing (Scherthan and Schönborn 2001), early DNA replication, centromere, and telomere effects, and an “intrinsic boost” (Wang *et al.* 2021).

Other factors, such as environmental temperature and genetic background, may affect the recombination pattern (Pazhayam *et al.* 2021). PRDM9 binds specifically to sequences localized at the center of HR hotspots, therefore, polymorphism in PRDM9-binding sites was supposed to affect the recombination frequency (Grey *et al.* 2018). *PRDM9*-A and *PRDM9*-C alleles were common in African populations, whereas only the *PRDM9*-A allele predominated in all non-African populations, implying the association between the *PRDM9*-C allele and the higher frequency of HR hotspots in African populations (Spence and Song 2019). However, the limitation of our microarray data did not allow the analysis of *PRDM9* allele frequency by this present data, which warrants further analysis.

From clinical point of view, the position of HR hotspots may benefit for improving accuracy in haplotype analysis for PGT-M. For example, Karyomapping, which is solely based on haplotyping, may be misdiagnosed due to recombination in the proband; in this condition, detection needs to be treated with caution. In our study, 6,494 (32.3%) hotspots were in genes of OMIM, which would pose a great challenge for PGT-M. Future clinical works should pay more attention to the impact of recombination, which may cause misdiagnosis of embryos. If recombination occurs in the mutation gene, the risk of misdiagnosis would be high. Direct mutation detection by Sanger sequence or PCR is essential in this situation.

Aneuploidy is the most important factor leading to pregnancy failure and early miscarriage. Whole chromosome aneuploidy is mainly due to meiotic errors in the oocytes. Due to the low aneuploidy rate in the sperm, most paternal chromosome errors are considered of mitotic origin. The location of crossovers might affect mis-segregation (Kong *et al.* 2004); for example, proximal recombination (Pazhayam *et al.* 2021) or crossovers near the centromere (Ottolini *et al.* 2015) might have an impact on the segregation of chromosomes (Nagaoka *et al.* 2012). The number of crossovers might also affect mis-segregation (Hassold *et al.* 2021). Trisomy 21 with an error in meiotic prophase I had fewer recombinations than their siblings (Middlebrooks *et al.* 2014). Also, the decreased number of recombination was associated with aneuploidy sperm (Ma *et al.* 2006). One study using fetal ovarian samples from elective terminations of pregnancies found that individuals with lower meiotic recombination rates were at an increased risk of producing aneuploid offspring (Hassold *et al.* 2021). Konstantinidis *et al.* (2020) analyzed the euploid chromosomes of aneuploidies and the same chromosomes of euploidies and discovered that embryos affected with either trisomy 16 only (*n* = 8) or trisomy 21 only (*n* = 5) had a lower incidence of recombination compared to euploid embryos but not in meiotic trisomy 22 only (*n* = 7). However, our results were discordant with the previous study. In this study, embryos affected with trisomy 22 only (*n* = 8, *P* = 0.036) had a higher incidence of recombination compared with euploid embryos but not in trisomy 16 only (*n* = 19, *P* = 0.659) or trisomy 21 only (*n* = 3, *P* = 0.059; Supplementary Table 6). Considering the small sample size, this conclusion needs to be treated with caution. Due to technical limitations, this study could not distinguish the recombination if both chromosomes enter the same gamete. Therefore, recombination events of the chromosomes specifically affected by aneuploidy were not evaluated. Further analysis based on other methods is needed in the future.

The mechanism of reciprocal translocation remains elusive. It was considered that DSB formation was the common basis for crossovers and reciprocal translocation (Gómez-Herreros 2019; Chen *et al.* 2020). HR repair formed crossovers, whereas NHEJ repair was liable to form rearrangement (Weinstock *et al.* 2006). Nonetheless, it was unclear what determined whether initial DSBs were processed by HR or the NHEJ repair subpathway. Transcription and genome organization were the 2 major determinants for DSBs and reciprocal translocation (Gómez-Herreros 2019). The prerequisite for translocation formation was a close spatial organization of the genome or structure (Roukos *et al.* 2013). In addition, illegitimate recombination events happened in palindromic AT-rich sequence mediating the constitutional *t*(11;22) translocation (Edelmann *et al.* 1999, 2001). Dai and Kong (2020) analyzed common chromosomal breakpoint regions among 586 carriers of reciprocal translocations and found the breakpoints occurred more in GC-rich sequences, which was associated with a high frequency of recombination and B-Z transformation of chromosome conformation (Collins *et al.* 1996). In this study, around 25% of breakpoints of reciprocal translocation overlapped with HR hotspots, suggesting both events may share a similar mechanism, likely due to DSBs.

This study has several advantages. First, 164 families including parents and 1,070 offspring were studied. Each pedigree constituted members ranging from 2 to 25, which was seldomly found in human populations, as only a few gametes per individual generate offspring. Therefore, more detailed information could be obtained from each pedigree. Second, HumanKaryomap-12, applied for PGT-M in this study, is particularly designed for monogenic diseases. It allowed the analysis of SNP data and recombination closely related to monogenic diseases, which may have clinical value for future applications. This study also provided the first genetic map for Han Chinese embryos. Lastly, our results supported the hypothesis that reciprocal translocation may occur more often in HR hotspots, as 25% of translocation breakpoints (*n* = 85) overlapped with HR hotspots in paternal and maternal genetic maps.

However, several limitations were also presented. First, the sample size and generation were small, which may introduce selection bias. The result of trophectoderm biopsy would not excellently represent the karyotypes of inner cell mass (Wu *et al.* 2021). WGA after trophectoderm biopsy may also bring bias. Controlled ovarian stimulation and ICSI would bypass natural selection and introduce bias. Last but not least, only a small part of hotspots (23.60% and 21.94% for paternal and maternal hotspots, respectively) overlapped with the previous study (Halldorsson *et al.* 2019). It may be mainly due to the small sample size and sparse marker set in this study. Ancestry differences may also contribute to the difference.

In conclusion, we found that HR occurred more often in maternal chromosomes, and the age effect was more significant in maternal HR. Part of the breakpoints of reciprocal translocations overlapped with HR hotspots, which implied a similar mechanism behind these events. Our study may provide a valuable basis for future genetic research, such as linkage studies, prediction of disease-causing structural rearrangements, and genome-wide association studies on the Han Chinese population.

## Supplementary Material

jkad031_Supplementary_Data

## Data Availability

The recombination data reported in this paper have been deposited in the OMIX, China National Center for Bioinformation/Beijing Institute of Genomics, Chinese Academy of Sciences (https://ngdc.cncb.ac.cn/omix: accession no. OMIX001077). Supplemental material is available at G3 online.
