# Supplementary material for: Mapping of meiotic recombination in human preimplantation blastocysts
Source: G3 (Bethesda). 2023 Feb 3;13(4):jkad031. doi: 10.1093/g3journal/jkad031 (PMC10085796; doi:10.1093/g3journal/jkad031)
Supplement: jkad031_Supplementary_Data [file jkad031_supplementary_data.zip › supplement legents.docx]

**Fig. S1 The characteristic of CNVs.**

Only aneuploidies with autosomal CNVs were analyzed. The CNV analysis wasn’t carried among sex chromosomes. The result of parent-of-origin analysis of segmental and whole chromosome aneuploidy was present as CNV origin. The result of mechanistic origin of aneuploidy was present as CNV happen time.

**Table S1. Marker and crossover data.**

**Table S2. The comparison of adjacent recombination events space between parental chromosomes.**

**Table S3. Hotspots and their related genes of Online Mendelian Inheritance in Man (OMIM).**

**Table S4. Comparison the recombination events between aneuploidy and euploidy in paternal chromosomes.**

**Table S5 Comparison the recombination events between aneuploidy and euploidy in maternal chromosomes.**

**Table S6 Comparison the recombination events between aneuploidy and euploidy.**
