# Supplementary material for: Mapping of meiotic recombination in human preimplantation blastocysts
Source: G3 (Bethesda). 2023 Feb 3;13(4):jkad031. doi: 10.1093/g3journal/jkad031 (PMC10085796; doi:10.1093/g3journal/jkad031)
Supplement: jkad031_Supplementary_Data [file jkad031_supplementary_data.zip › Table S5.docx]

**Table S5 Comparison the recombination events between aneuploidy and euploidy in maternal chromosomes.**

| chr | aneuploidy | | Euploidy | | *p* |
| --- | --- | --- | --- | --- | --- |
|  | total | recombination | total | Recombination |  |
| 1 | 325 | 3.86±1.73 | 910 | 3.69±1.76 | 0.135 |
| 2 | 327 | 3.45±1.64 | 910 | 3.30±1.60 | 0.164 |
| 3 | 333 | 2.87±1.51 | 910 | 2.97±1.51 | 0.274 |
| 4 | 329 | 2.84±1.42 | 910 | 2.86±1.52 | 0.874 |
| 5 | 323 | 2.83±1.52 | 910 | 2.71±1.46 | 0.209 |
| 6 | 327 | 2.63±1.52 | 910 | 2.53±1.41 | 0.274 |
| 7 | 320 | 2.55±1.51 | 910 | 2.25±1.33 | 0.001 |
| 8 | 329 | 2.38±1.28 | 910 | 2.17±1.28 | 0.011 |
| 9 | 331 | 2.15±1.26 | 910 | 2.04±1.30 | 0.199 |
| 10 | 327 | 2.22±1.21 | 910 | 2.26±1.27 | 0.609 |
| 11 | 332 | 2.03±1.21 | 910 | 2.00±1.21 | 0.740 |
| 12 | 330 | 2.39±1.33 | 910 | 2.15±1.24 | 0.003 |
| 13 | 333 | 1.69±1.04 | 910 | 1.62±1.04 | 0.265 |
| 14 | 337 | 1.57±1.08 | 910 | 1.46±1.01 | 0.087 |
| 15 | 318 | 1.77±1.11 | 910 | 1.61±1.07 | 0.017 |
| 16 | 291 | 1.87±1.18 | 910 | 1.73±1.16 | 0.063 |
| 17 | 326 | 1.89±1.13 | 910 | 1.72±1.11 | 0.018 |
| 18 | 329 | 1.74±1.10 | 910 | 1.56±1.05 | 0.006 |
| 19 | 332 | 1.46±1.03 | 910 | 1.30±0.93 | 0.007 |
| 20 | 334 | 1.49±1.00 | 910 | 1.37±0.95 | 0.050 |
| 21 | 325 | 0.77±0.73 | 910 | 0.74±0.72 | 0.543 |
| 22 | 315 | 0.94±0.86 | 910 | 0.88±0.78 | 0.293 |
| X and Y | 329 | 2.09±1.27 | 910 | 1.98±1.23 | 0.191 |

Chr, chromosome

Each chromosome in aneuploidy represents the euploid chromosome.

Only the family with both aneuploidy and euploidy was included in analysis for recombination in euploidy.
