# Supplementary material for: Mapping of meiotic recombination in human preimplantation blastocysts
Source: G3 (Bethesda). 2023 Feb 3;13(4):jkad031. doi: 10.1093/g3journal/jkad031 (PMC10085796; doi:10.1093/g3journal/jkad031)
Supplement: jkad031_Supplementary_Data [file jkad031_supplementary_data.zip › Table_S1_G3-2022-403707.docx]

**Table S1. Marker and crossover data.**

| Type of data | Paternal | Maternal | Autosomal | Chr X | Total |
| --- | --- | --- | --- | --- | --- |
| Microarray markers | - | - | 277,128 | 15,981 | 293,109 |
| Meiosis | 1,070 | 1,070 | - | - | 2,140 |
| Crossovers | 31,005 | 49,466 | 77,774 | 2,697 | 80,471 |

Chr X, chromosome X
