# Supplementary material for: Mapping of meiotic recombination in human preimplantation blastocysts
Source: G3 (Bethesda). 2023 Feb 3;13(4):jkad031. doi: 10.1093/g3journal/jkad031 (PMC10085796; doi:10.1093/g3journal/jkad031)
Supplement: jkad031_Supplementary_Data [file jkad031_supplementary_data.zip › Table_S2_G3-2022-403707.docx]

**Table S2 The comparison of adjacent recombination events space between parental chromosomes.**

| Chr. | total | min | max | mean | SD |  | Paternal | | |  | Maternal | | | *p* |
| --- | --- | --- | --- | --- | --- | --- | --- | --- | --- | --- | --- | --- | --- | --- |
|  |  |  |  |  |  |  | total | mean | SD |  | total | mean | SD |  |
| 1 | 4188 | 35177 | 243594554 | 66615928.37 | 50950072.267 |  | 1301 | 90635734.52 | 57079218.54 |  | 2887 | 55791623.62 | 43832178.06 | ＜0.001 |
| 2 | 3576 | 23109 | 239465293 | 70049101.61 | 49075671.234 |  | 1106 | 94354381.83 | 54814766.05 |  | 2470 | 59165846.58 | 41945564.57 | ＜0.001 |
| 3 | 2971 | 96613 | 195959512 | 61633751.31 | 43357005.902 |  | 890 | 84222515.07 | 48297607.59 |  | 2081 | 51973011.4 | 37087586.1 | ＜0.001 |
| 4 | 2641 | 198549 | 188478069 | 61042969.59 | 42440532.694 |  | 649 | 91996770.78 | 46747688.4 |  | 1992 | 50958121.72 | 35538720.52 | ＜0.001 |
| 5 | 2554 | 52669 | 176656431 | 59371497.62 | 39605649.818 |  | 710 | 84867909.57 | 44436713.25 |  | 1844 | 49554549.42 | 32655902.5 | ＜0.001 |
| 6 | 2342 | 29206 | 168042553 | 58802959.03 | 40042916.192 |  | 684 | 81896651.03 | 45714009.12 |  | 1658 | 49275766.43 | 33060048.86 | ＜0.001 |
| 7 | 2140 | 42399 | 154827368 | 55399044.32 | 35700011.243 |  | 697 | 71040436.04 | 39626723.68 |  | 1443 | 47843916.1 | 30939218.91 | ＜0.001 |
| 8 | 1782 | 48397 | 142472491 | 53209618.15 | 34732883.775 |  | 450 | 78568064.17 | 38020800.1 |  | 1332 | 44642575.57 | 28908918.96 | ＜0.001 |
| 9 | 1723 | 176999 | 138147055 | 56275507.13 | 36183948.265 |  | 552 | 74825987.23 | 40486814.26 |  | 1171 | 47530959.72 | 30267894.46 | ＜0.001 |
| 10 | 1991 | 329371 | 131853959 | 48625705.86 | 30706195.998 |  | 576 | 66880716.21 | 34598244.29 |  | 1415 | 41194691.04 | 25478857.65 | ＜0.001 |
| 11 | 1739 | 132750 | 133626973 | 53154692.39 | 33197001.621 |  | 588 | 70019280.15 | 37280691.78 |  | 1151 | 44539247.04 | 27136299.21 | ＜0.001 |
| 12 | 1934 | 98527 | 131457029 | 50534539.20 | 31821452.158 |  | 674 | 65072419.63 | 36669165.42 |  | 1260 | 42757926.97 | 25734352.23 | ＜0.001 |
| 13 | 1197 | 15435 | 94109236 | 42938653.28 | 23743049.017 |  | 397 | 52249837.64 | 25964842.99 |  | 800 | 38317978.04 | 21100857.61 | ＜0.001 |
| 14 | 938 | 257828 | 82091163 | 38209867.30 | 21293490.157 |  | 278 | 48041520.32 | 21893774.28 |  | 660 | 34068655.88 | 19627821.15 | ＜0.001 |
| 15 | 1159 | 108197 | 78961634 | 32211306.06 | 19923040.918 |  | 380 | 40073929.50 | 23424125.230 |  | 779 | 28375879.99 | 16690796.960 | ＜0.001 |
| 16 | 1254 | 37492 | 88051827 | 37620871.27 | 23570299.016 |  | 347 | 55800749.77 | 25665246.16 |  | 907 | 30665614.55 | 18486932.45 | ＜0.001 |
| 17 | 1383 | 29617 | 79855326 | 33294817.56 | 21119661.834 |  | 502 | 43991963.41 | 25160112.7 |  | 881 | 27199508.58 | 15414863.02 | ＜0.001 |
| 18 | 1054 | 63276 | 76933248 | 33574276.94 | 20493037.980 |  | 316 | 45373829.41 | 23668698.16 |  | 738 | 28521894.04 | 16593239.1 | ＜0.001 |
| 19 | 952 | 263838 | 58394099 | 28056761.29 | 16453199.706 |  | 433 | 33155223.7 | 19791124.92 |  | 519 | 23803130.8 | 11422697.44 | ＜0.001 |
| 20 | 927 | 329796 | 61499862 | 30556584.27 | 17070841.076 |  | 353 | 39572071.46 | 18586389.78 |  | 574 | 25012216.71 | 13336364.17 | ＜0.001 |
| 21 | 214 | 45284 | 27961502 | 15013036.30 | 7835780.320 |  | 61 | 9284752.636 | 7316273.563 |  | 153 | 17296861.81 | 6818501.499 | ＜0.001 |
| 22 | 346 | 182385 | 32534360 | 16034137.02 | 8415170.860 |  | 97 | 18216291.25 | 9224134.992 |  | 249 | 15184060.88 | 7936556.063 | 0.005 |
| Chr, chromosome; SD, standard deviation  We used middle SNP represent the location of recombination event. The spaces between adjacent recombination events were defined as the spaces between adjacent recombination middle SNPs. | | | | | | | | | | | | | | |
