# Supplementary material for: Mapping of meiotic recombination in human preimplantation blastocysts
Source: G3 (Bethesda). 2023 Feb 3;13(4):jkad031. doi: 10.1093/g3journal/jkad031 (PMC10085796; doi:10.1093/g3journal/jkad031)
Supplement: jkad031_Supplementary_Data [file jkad031_supplementary_data.zip › Table_S4_G3-2022-403707.docx]

**Table S4 Comparison the recombination events between aneuploidy and euploidy in paternal chromosomes.**

| Chr | aneuploidy | | euploidy | | *p* |
| --- | --- | --- | --- | --- | --- |
|  | total | recombination | total | recombination |  |
| 1 | 325 | 2.35±1.31 | 910 | 2.17±1.22 | 0.027 |
| 2 | 327 | 2.18±1.15 | 910 | 1.99±1.13 | 0.012 |
| 3 | 333 | 1.79±1.01 | 910 | 1.70±1.08 | 0.207 |
| 4 | 329 | 1.62±1.04 | 910 | 1.48±0.92 | 0.039 |
| 5 | 323 | 1.59±1.03 | 910 | 1.53±1.01 | 0.398 |
| 6 | 327 | 1.63±0.94 | 910 | 1.51±1.00 | 0.054 |
| 7 | 320 | 1.69±1.01 | 910 | 1.53±1.03 | 0.018 |
| 8 | 329 | 1.34±0.90 | 910 | 1.20±0.88 | 0.017 |
| 9 | 331 | 1.59±0.98 | 910 | 1.33±0.93 | <0.001 |
| 10 | 327 | 1.47±0.97 | 910 | 1.37±0.90 | 0.081 |
| 11 | 332 | 1.49±0.99 | 910 | 1.40±0.93 | 0.106 |
| 12 | 330 | 1.56±0.99 | 910 | 1.47±1.02 | 0.169 |
| 13 | 333 | 1.07±0.83 | 910 | 1.17±0.82 | 0.052 |
| 14 | 337 | 1.11±0.83 | 910 | 0.99±0.77 | 0.019 |
| 15 | 318 | 1.16±0.80 | 910 | 1.13±0.87 | 0.581 |
| 16 | 291 | 1.25±0.86 | 910 | 1.12±0.80 | 0.017 |
| 17 | 326 | 1.23±0.86 | 910 | 1.30±0.92 | 0.260 |
| 18 | 329 | 1.06±0.79 | 910 | 1.02±0.82 | 0.387 |
| 19 | 332 | 1.22±0.89 | 910 | 1.18±0.87 | 0.490 |
| 20 | 334 | 1.19±1.83 | 910 | 1.08±0.83 | 0.039 |
| 21 | 325 | 0.66±0.62 | 910 | 0.56±0.61 | 0.022 |
| 22 | 315 | 0.72±0.65 | 910 | 0.67±0.65 | 0.215 |
| X and Y | 329 | 0.76±0.68 | 910 | 0.6±0.63 | <0.001 |

Chr, chromosome

Each chromosome in aneuploidy represents the euploid chromosome.

Only the family with both aneuploidy and euploidy was included in analysis for recombination in euploidy.
