# Supplementary material for: Mapping of meiotic recombination in human preimplantation blastocysts
Source: G3 (Bethesda). 2023 Feb 3;13(4):jkad031. doi: 10.1093/g3journal/jkad031 (PMC10085796; doi:10.1093/g3journal/jkad031)
Supplement: jkad031_Supplementary_Data [file jkad031_supplementary_data.zip › Table_S6_G3-2022-403707.docx]

**Table S6 Comparison the recombination events between aneuploidy and euploidy.**

|  | aneuploidy | |  | euploidy | | *P*-value |
| --- | --- | --- | --- | --- | --- | --- |
|  | total | recombination |  | total | recombination |  |
| Trisomy 16 only | 19 | 74.21±8.28 |  | 141 | 75.84±15.78 | 0.659 |
| Trisomy 21 only | 3 | 92.00±11.53 |  | 15 | 75.13±13.16 | 0.059 |
| Trisomy 22 only | 8 | 79.88±10.02 |  | 48 | 71.10±10.75 | 0.036 |
