# Supplementary figures and images for: Mapping of meiotic recombination in human preimplantation blastocysts
Source: G3 (Bethesda). 2023 Feb 3;13(4):jkad031. doi: 10.1093/g3journal/jkad031 (PMC10085796; doi:10.1093/g3journal/jkad031)

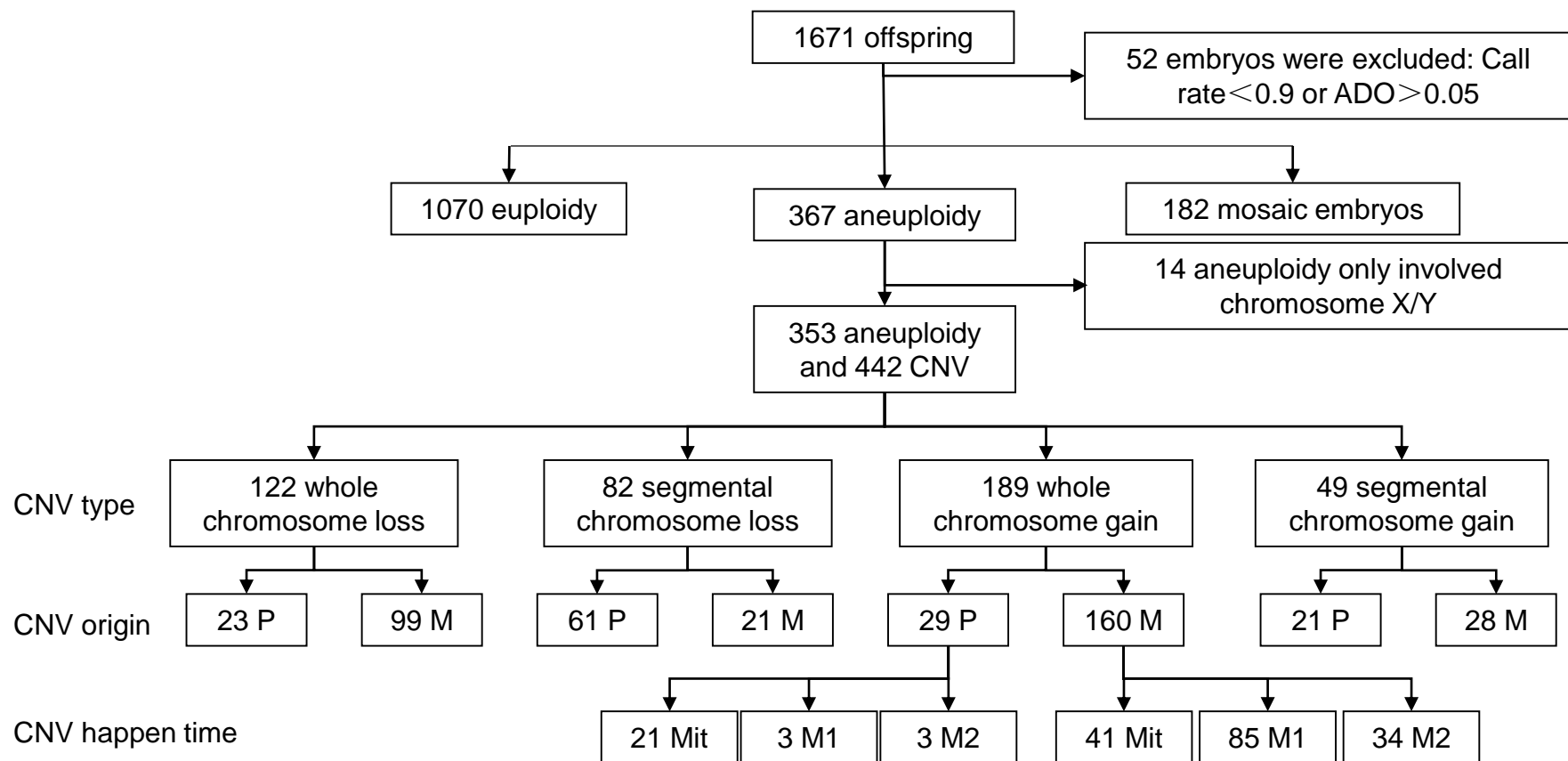

Supplement: jkad031_Supplementary_Data [file jkad031_supplementary_data.zip › Figure_S1_G3-2022-403707.pdf]
